# First person – Kriti Chaplot

**DOI:** 10.1242/dmm.038984

**Published:** 2019-02-07

## Abstract

First Person is a series of interviews with the first authors of a selection of papers published in Disease Models & Mechanisms, helping early-career researchers promote themselves alongside their papers. Kriti Chaplot is first author on ‘SOD1 activity threshold and TOR signalling modulate VAP(P58S) aggregation via reactive oxygen species-induced proteasomal degradation in a *Drosophila* model of amyotrophic lateral sclerosis’, published in DMM. Kriti is a PhD student in the lab of Dr Girish Ratnaparkhi at the Indian Institute of Science Education and Research, Pune, India. Her main research interest is delineating cellular mechanisms that perturb aggregation in neurodegenerative diseases.


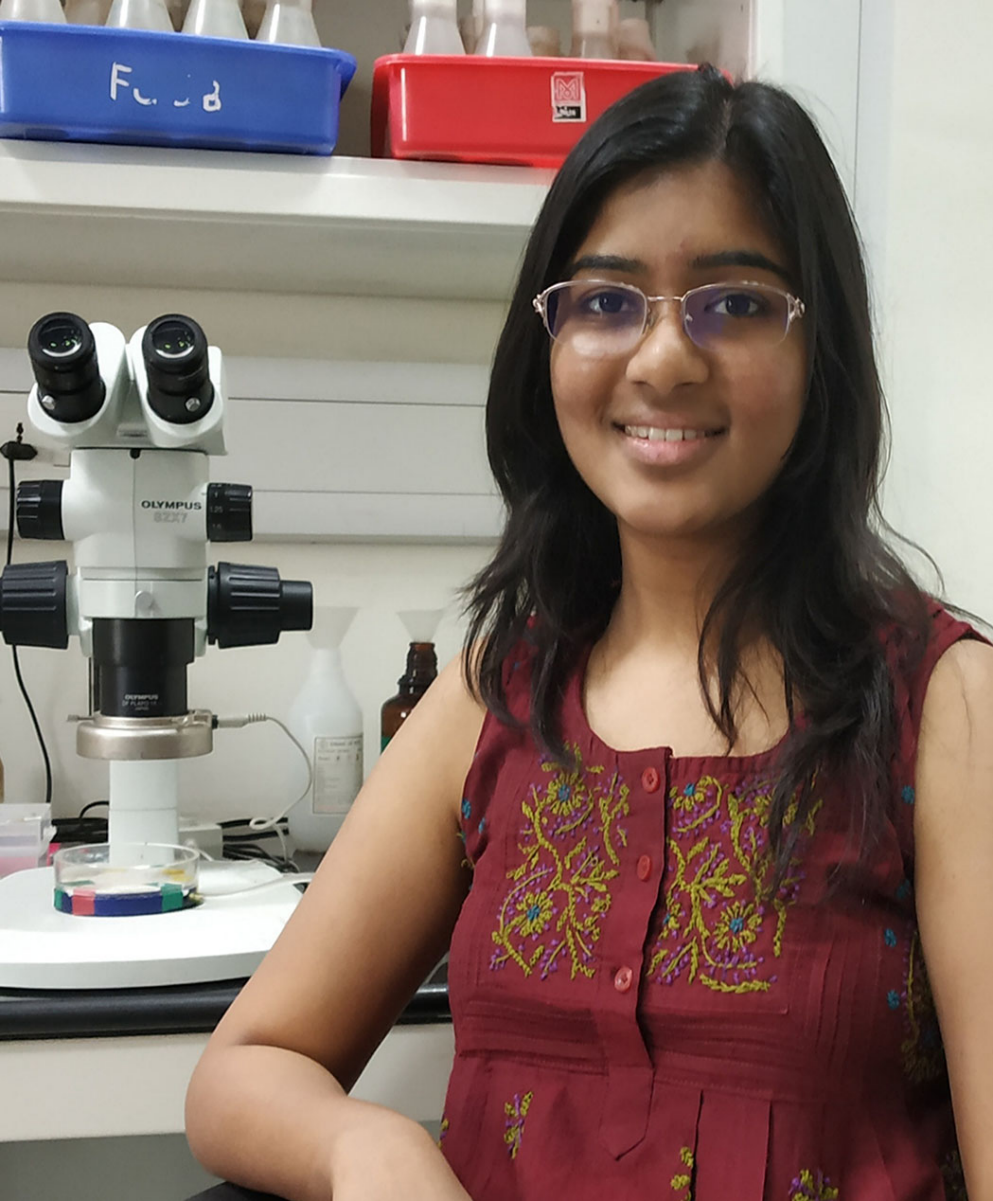


**Kriti Chaplot**

**How would you explain the main findings of your paper to non-scientific family and friends?**

I study mutated genes and proteins that cause Lou Gehrig's disease, also known as amyotrophic lateral sclerosis (ALS). The disease is caused by the death of neurons that control muscle movement. In the disease, some mutated proteins in the brain become unstable and clump together to form ‘aggregates’. Presence of these aggregates can affect disease progression. The neurons can then engage processes to remove the mutant protein aggregates in order to prevent further damage to the brain. In my study, I have identified one such process that acts as a garbage disposal machine in the neuron, helping to reduce a particular kind of mutant protein aggregate. I perform my studies in the brain of a fruit fly as a mimic to understand human brain disease.

“[…] I have identified one such process that acts as a garbage disposal machine in the neuron, helping to reduce a particular kind of mutant protein aggregate.”

**What are the potential implications of these results for your field of research?**

The study highlights a functional interaction between two disease-causing loci in ALS, namely *Sod1* and *VAP*. Our study serves as an example of how aggregates of one protein (mutant VAP) are regulated by the other (SOD1 activity). We identify a role for a threshold level of reactive oxygen species (ROS), mediated by both SOD1 levels and mTOR signalling, in activating the ubiquitin proteasomal system (UPS), in order to clear ALS8 mutant aggregates. Curiously, ROS are often found to be upregulated in neurodegenerative disorders. According to our hypothesis, if ROS can be pharmacologically modulated in the diseased cell, they could, in turn, mediate degradative mechanisms to clear mutated proteins, thereby influencing disease progression. Thus, ROS could serve as a potential therapeutic target in ALS. Indeed, edavarone is a recently approved ALS drug that works by altering the levels of ROS. Rapamycin, an inhibitor of the mTOR pathway, is also in phase-III trials for ALS.

**What are the main advantages and drawbacks of the model system you have used as it relates to the disease you are investigating?**

I have used *Drosophila melanogaster* as a model system to study ALS. Most of the disease-causing genes in ALS have orthologues in *Drosophila.* Flies serve as an excellent animal system to perform genetic and pharmacological screens as performed in our study. The availability of resources from agencies such as Bloomington *Drosophila* Stock Center and *Drosophila* Genome Research Center have been instrumental in aiding genetic studies. The *Drosophila* nervous system has been extensively used to model neurodegenerative disorders. We were able to use the larval brain to overexpress and study VAP mutant protein aggregates, and cellular functions that modulate its levels using genetic and pharmacological perturbations. Owing to functional similarities with the human spinal cord, the ventral nerve cord of the larval brain provided a simple system in which neuron-specific signalling pathways could be deciphered, thus establishing genetic interactions. Flies are easy to rear and allow for testing of a large sample size to affirm our results. However, a key drawback of our model system is that studies performed in invertebrates, such as flies, cannot be extrapolated to humans without validation using vertebrate systems. Moreover, fly models to date (developed by us and others) only mimic a few phenotypes associated with complex neurodegenerative disorders, but not the disease itself.

**What has surprised you the most while conducting your research?**

ROS is known to increase misfolding and subsequent aggregation of mutant proteins in neurodegenerative diseases as seen in the case of tau, beta-amyloid, SOD1 etc. The most surprising part of our study was that, contrary to popular notions, we found that ROS could decrease VAP mutant protein aggregation.

**Figure:**
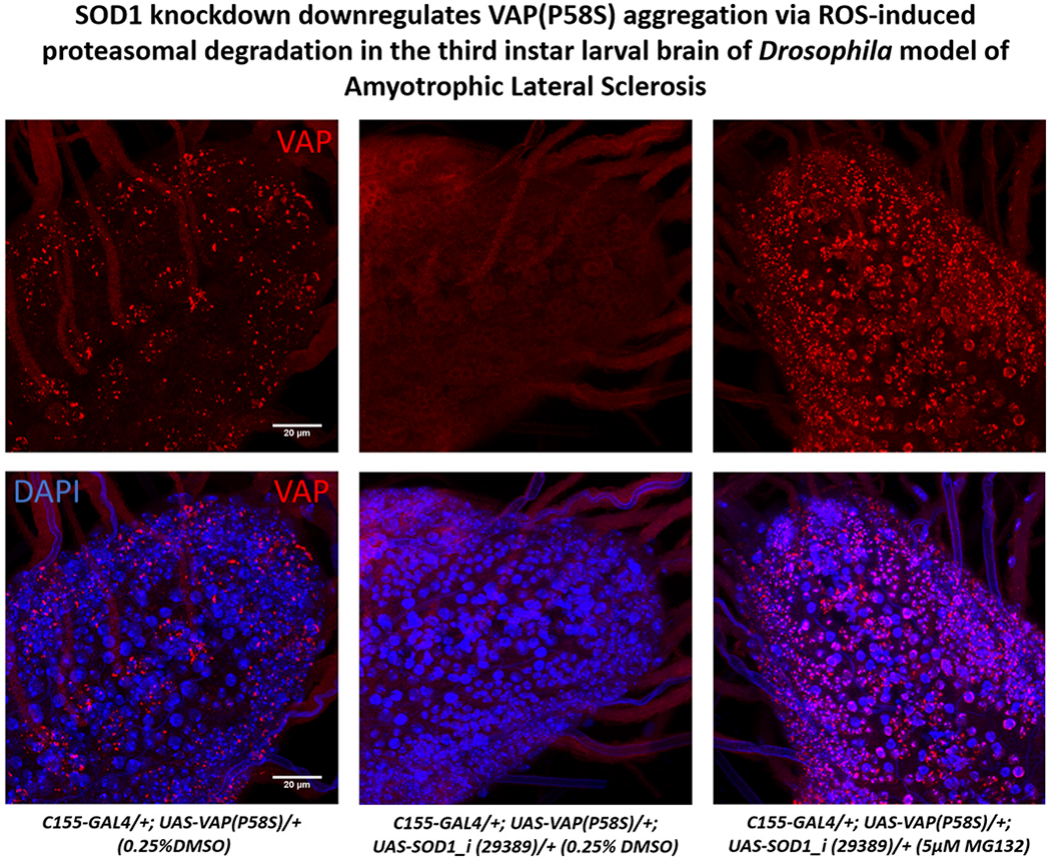
**ROS-induced proteasomal degradation of VAP(P58S) aggregates.**

**Describe what you think is the most significant challenge impacting your research at this time and how will this be addressed over the next 10 years?**

The challenges impacting my research at this time are twofold. In our study, we use the overexpression of VAP mutant in order to study aggregation in the *Drosophila* model system. While it is useful in identifying modulators of aggregation and neurodegenerative phenotypes, is not a true mimic of the disease as a whole. With the advent of the genome-editing tools, in particular, the CRISPR technology, these models may be greatly improved to represent the disease condition. The other challenge is the lack of understanding of a common mechanism in the disease. Genome-wide studies have helped identify over 50 potential loci in ALS; however, a common therapeutic target remains elusive and ALS remains an incurable disease. Attempts to find personalized treatment options for patients of similar genetic and pathological subgroups may help combat the disease.

**What changes do you think could improve the professional lives of early-career scientists?**

In India, there is a dearth of postdoc-driven science, where most of the work force consists of PhD students. This has led to a large number of PhDs in the country with uncertain job opportunities to continue in academia. This is coupled with the major issue of insufficient number of grants and funding for early-career scientists. Although various government-approved fellowships for postdoctoral studies have been announced, they do not ensure job stability or tenured positions after the fellowship period. There is a need to develop more streamlined programmes to address this matter. Inadequate access to cutting-edge technology and infrastructure is another problem faced by Indian scientists. Collaborative programs for early-career scientists at a global level could solve some of these issues.

**What's next for you?**

I am keen on working with human patient samples to further delineate alterations in cellular processes that manifest in ALS pathogenesis. With an extensive background in genetics and ALS fly models, I want to pursue my postdoctoral studies focusing on translational research in neurodegenerative disorders.

“[…] we still need more gender-neutral policies to further create equal opportunities for men and women for scientific as well as administrative positions based on qualifications and competence, as opposed to preconceived or societal definitions.”

**How have opportunities for women in science changed over the years?**

I feel there has been a substantial improvement in opportunities offered to women in science over the last few decades. In recent years, there has been an increase in awareness in scientific circles at a global level. However, we still need more gender-neutral policies to further create equal opportunities for men and women for scientific as well as administrative positions based on qualifications and competence, as opposed to preconceived or societal definitions.
